# Comparisons of subunit 5A and 5B isoenzymes of yeast cytochrome *c* oxidase

**DOI:** 10.1042/BJ20140732

**Published:** 2014-12-05

**Authors:** Raksha Dodia, Brigitte Meunier, Christopher W. M. Kay, Peter R. Rich

**Affiliations:** *Institute of Structural and Molecular Biology, University College London, Gower Street, London WC1E 6BT, U.K.; †Centre de Génétique Moléculaire du CNRS, UPR 3404, avenue de la Terrasse, Gif-sur-Yvette Cedex 91198, France; ‡London Centre for Nanotechnology, University College London, 17–19 Gordon Street, London WC1H 0AH, U.K.

**Keywords:** complex IV, cytochrome *c* oxidase, Michaelis–Menten constant, oxygen affinity, subunit 5 isoform, turnover number, C*c*O, cytochrome *c* oxidase, cyt *c*, cytochrome *c*, mitos, mitochondrial membranes, DDM, *n*-dodecyl β-D-maltoside, TMPD, *N*,*N*,*N*′,*N*′-tetramethyl-*p*-phenylenediamine, CCCP, carbonyl cyanide *m*-chlorophenylhydrazone

## Abstract

Subunit 5 of *Saccharomyces cerevisiae* cytochrome *c* oxidase (C*c*O) is essential for assembly and has two isoforms, 5A and 5B. 5A is expressed under normoxic conditions, whereas 5B is expressed at very low oxygen tensions. As a consequence, *COX5A*-deleted strains (Δ*cox5A*) have no or only low levels of C*c*O under normoxic conditions rendering them respiratory deficient. Previous studies have reported that respiratory growth could be restored by combining Δ*cox5A* with mutations of *ROX1* that encodes a repressor of *COX5B* expression. In these mutants, 5B isoenzyme expression level was 30–50% of wild-type (5A isoenzyme) and exhibited a maximum catalytic activity up to 3-fold faster than that of 5A isoenzyme. To investigate the origin of this effect, we constructed a mutant strain in which *COX5B* replaced *COX5A* downstream of the *COX5A* promoter. This strain expressed wild-type levels of the 5B isoenzyme, without the complication of additional effects caused by mutation of *ROX1*. When produced this way, the isoenzymes displayed no significant differences in their maximum catalytic activities or in their affinities for oxygen or cytochrome *c*. Hence the elevated activity of the 5B isoenzyme in the *rox1* mutant is not caused simply by exchange of isoforms and must arise from an additional effect that remains to be resolved.

## Abstract 2

Yeast cytochrome *c* oxidase isoenzymes with subunits 5A or 5B can exhibit different kinetic properties. We demonstrate that these differences are not induced by simple 5A/5B replacement and that the effect additionally requires expression level changes and/or undefined post-translational effects.

## INTRODUCTION

Mitochondrial C*c*O (cytochrome *c* oxidase) is the terminal enzyme of the respiratory chain that catalyses electron transfer from cyt *c* (cytochrome *c*) to oxygen. The reaction is coupled to the translocation of protons across the mitochondrial inner membrane, forming a protonmotive force used to drive ATP synthesis and giving an overall reaction of: 4 cyt *c*^2+^+8 H^+^_mitochondrial matrix_ + O_2_→4 cyt *c*^3+^+4 H^+^_intermembrane space_+2 H_2_O. *Saccharomyces cerevisiae* C*c*O is composed of at least 11 subunits. Subunits I, II and III are encoded by the mitochondrial genome and constitute the essential catalytic core where the metal centres Cu_A_ (subunit II), haem *a*, haem *a*_3_ and Cu_B_ (subunit I), the hydrophilic pathways for proton transfer (subunit I) and the oxygen entrance pathway (subunit III) are located. These subunits are conserved across eukaryotic and prokaryotic homologues.

The remaining eight supernumerary subunits, which do not have bacterial homologues, are encoded by the nuclear genome. They are homologous with eight of the ten supernumerary subunits of human/bovine C*c*O [1–3]. Some of these mammalian supernumerary subunits are involved in assembly, dimerization or stability. Others may provide means of regulation by allosteric control, for example via ligand-binding [4–6] or phosphorylation sites [7,8].

Five of the mammalian supernumerary subunits display isoforms that are differentially expressed depending on the tissue type or stage of development [6,9,10]. Subunit IV, the largest of the supernumerary subunits (~17 kDa), is of particular interest. It has a single membrane spanning α-helix that is closely associated with helices 11 and 12 of core subunit I. A hydrophilic C-terminal domain projects into the intermembrane space close to the cyt *c*-binding site on subunit II, and a hydrophilic N-terminal domain projects into the matrix [11]. ATP/ADP-binding sites are predicted on both matrix [4] and cytosolic [5] domains that can allosterically inhibit C*c*O activity by exchanging bound ADP for ATP at high ATP/ADP ratios, hence providing a negative-feedback loop mechanism of respiratory control [12–14]. It was reported that cAMP-dependent phosphorylation of Ser^58^, located in the matrix domain of subunit IV, modulates the respiratory activity by controlling ATP allosteric inhibition [15]. This serine residue is conserved in mammalian C*c*Os but not in other species. Furthermore, subunit IV has two isoforms: IV-2 is predominantly expressed in lung, neurons and fetal muscle, whereas IV-1 is expressed across all tissue types [16,17]. IV-2 expression has been shown to be induced by hypoxic conditions under the control of RBPJ (recombination signal-binding protein for immunoglobulin κJ region), CXXC5 and CHCHD2 (coiled-coil-helix-coiled-coil-helix domain containing 2) transcription factors [18] or by toxins [19]. The IV-2 isoenzyme from lung has been reported to have a faster turnover activity than the IV-1 isoenzyme from liver [12]. In addition, IV-2 expression abolishes the allosteric inhibition by ATP [6,16].

Subunit 5 of yeast C*c*O is homologous with mammalian subunit IV [1] and also displays two isoforms (5A and 5B) [20,21] that are encoded by single-copy genes: *COX5A* on chromosome 14 and *COX5B* on chromosome 9. They share 67% nucleotide (66% amino acid) identity and are thought to have arisen from gene duplication followed by sequence divergence 130 million years ago [21]. One or other polypeptide is essential for C*c*O assembly. Structurally, they are superimposable on the crystal structure of subunit IV of bovine C*c*O [3,22]. However, matching of specific isoforms to those of mammalian subunit IV (5A with IV-1/IV-2 and 5B with IV-2/IV-1) is not possible based on the modest sequence identity of ~20% for each combination [3]. As with the mammalian subunit IV isoforms, the expression pattern of subunits 5A and 5B is controlled by oxygen (and haem) concentration [23,24]. Subunit 5A is expressed above 1 μM O_2_ and subunit 5B under low (<1 μM O_2_) oxygen concentrations [25]. Oxygen stimulates haem synthesis that then binds to and activates the transcription inducer Hap2/3/4/5 complex of *COX5A*; haem also activates Hap1 that induces the expression of Rox1, a repressor of *COX5B* expression. Under low oxygen, haem levels fall and Hap1 and Hap2/3/4/5 are no longer activated. This prevents *COX5A* transcription and loss of Rox1 transcription allows derepression of a set of hypoxic genes including *COX5B* [26,27].

Since subunit 5A or 5B is required for C*c*O assembly, C*c*O is not assembled or is at very low levels in *COX5A-*deleted strains at normal oxygen levels. If additionally the *rox1* gene is mutated, the *COX5B* gene is expressed at normal oxygen levels, resulting in an assembled 5B isoenzyme [24]. However, the C*c*O level was lower by a factor of 2–3 compared with the control *COX5A*-expressing strain. Intriguingly, it was observed that the 5B isoenzyme had a turnover number that was up to 3-fold faster than that of the 5A isoenzyme [28–30]. This was linked to an increased electron transfer rate from haem *a* to *a*_3_ *in vivo*. The subunit 5 isoforms were proposed to mediate their effects by allosterically altering the protein environment around haems *a* and *a*_3_ [28,29]. Infrared studies on CO bound C*c*O also indicated that the 5A isoform had two interchangeable conformers (CI and CII) whereas the 5B isoform had only the CII conformer. This led to the hypothesis that the CII conformer was capable of faster turnover activity [28].

In order to investigate the properties of the 5A and 5B isoenzymes more directly, we constructed a mutant strain expressing only subunit 5B by replacing the *COX5A* open reading frame with *COX5B* so that it was under the control of the *COX5A* promoter. This mutant expressed the 5B isoenzyme at normal levels under aerobic growth conditions, and allowed comparisons of the catalytic properties of the isoenzymes without the complication of low expression levels or of other possible changes caused by mutations in the transcription factor *Rox1*. As a further control, a mutant equivalent to that previously reported was constructed in which *COX5B* expression was achieved through deletion of *ROX1* in a *COX5A*-deleted strain and thereby allowed us to compare 5A and 5B isoenzyme kinetics in a controlled manner.

## MATERIALS AND METHODS

Chemicals were purchased from Sigma–Aldrich. Yeast iso-1 cyt *c* and iso-2 cyt *c* were a gift from B. Guiard (CGM, CNRS, Gif-sur-Yvette, France).

### Yeast strains

All strains (Table 1) were constructed from a modified *S. cerevisiae* strain W303-1B (α*ade2 HIS3 leu2 trp1 ura3*) that expressed wild-type C*c*O with a His_6_ tag sequence attached to *Cox13* [31]. Deletion and gene replacement were performed via homologous recombination of PCR products. The PCR products and gene replacements were confirmed by DNA sequencing. The plasmid pRS415-COX5A containing *COX5A* gene under the control of its promoter and terminator was a gift from D. Winge (University of Utah, Salt Lake City, UT, U.S.A.).

**Table 1:** *Saccharomyces cerevisiae* strains used.

Strain	Genotype	Description and respiratory growth phenotype
WTCOX5A	*COX5A*	Expression of *COX5A* under normal aerobic growth conditions. Respiratory growth competent
ΔCOX5A	*cox5a::URA3*	Deletion of *COX5A*. Respiratory growth deficient
ΔCOX5ApCOX5A	*cox5a::URA3*, *pCOX5A*	*COX5A* cloned on a centromeric plasmid under the control of its own promoter. Deletion of genomic *COX5A*. Respiratory growth competent
COX5B	*cox5a::COX5B*	Replacement of *COX5A* by *COX5B* downstream of the *COX5A* promoter on the nuclear genome. Expression of *COX5B* under normal aerobic growth conditions. Respiratory growth competent
pCOX5B	*cox5a::URA3, pcox5a::COX5B*	*COX5B* cloned downstream of *COX5A* promoter on a centromeric plasmid. Deletion of genomic *COX5A*. Respiratory growth competent
ΔROX1ΔCOX5A	*rox1::kanMX4*, *cox5a::URA3*	Up-regulation of *COX5B* through deletion of its transcription repressor *ROX1* in *COX5A*-deleted background. Weak respiratory growth
ΔROX1ΔCOX5ApCOX5A	*rox1::kanMX4*, *cox5a::URA3*, *pCOX5A*	*COX5A* with its own promoter on a centromeric plasmid. Deletion of *ROX1*, the transcription repressor of *COX5B*. Respiratory growth competent
ΔROX1ΔCOX5ApCOX5B	*rox1::kanMX4*, *cox5a::URA3*, *pcox5a::COX5B*	*COX5B* cloned downstream of the *COX5A* promoter on a centromeric plasmid. Deletion of *ROX1*, the transcription repressor of *COX5B*, in *COX5A*-deleted background. Respiratory growth competent

### Growth conditions and mitochondrial membrane preparation

Respiratory growth competence was checked on YPG medium (1% yeast extract, 2% peptone, 2% glycerol and 2% agar). All strains were grown in YPGal (1% yeast extract, 2% peptone and 2% galactose) and their mitos (mitochondrial membranes) were prepared by published protocols [31] and stored in 50 mM potassium phosphate and 2 mM EDTA, pH 7.4. Concentrations of C*c*O were measured from sodium dithionite reduced minus oxidized difference spectra at 605–621 nm with an absorption coefficient, Δ*ε*, of 26 mM^−1^·cm^−1^ or at 445–465 nm with a Δ*ε* of 204 mM^−1^·cm^−1^ (based on values for bovine C*c*O [32]). Protein contents were determined using the Bradford assay [33].

### Preparation of cyt *c*

Horse heart, yeast iso-1 cyt *c* (isoform-1) and iso-2 cyt *c* (isoform-2) stock solutions were prepared by washing with 20× volume of the reaction buffer (10 mM potassium phosphate, pH 6.6, and 50 mM KCl) using a 10 kDa Vivaspin centrifugal concentrator at 4°C and 10000 ***g***. The cyt *c* concentrations were measured from visible absorption spectra of sodium dithionite reduced samples, using an ε of 27.7 mM^−1^·cm^−1^ at 550 nm [34].

### Determination of oxygen affinity

The *K*_m_ of C*c*O for oxygen was measured using the myoglobin method [35]. Oxygen consumption rates at low oxygen concentrations were monitored by following the conversion of oxymyoglobin into myoglobin at 582–564 nm using a Shimadzu dual-wavelength spectrophotometer. Oxygenated myoglobin was prepared by reduction with sodium ascorbate, followed by separation into aerobic 10 mM potassium phosphate (pH 6.6) and 50 mM KCl with a Sephadex G-25 column (15 cm×1 cm). The total myoglobin concentration was measured from a visible absorption spectrum of a dithionite reduced sample, using an ε of 12.92 mM^−1^·cm^−1^ at 555 nm [36] and samples were typically 85% in the oxyferrous form and 15% in the ferric form. The reaction was carried out in a stoppered cuvette with stirring at 25°C. The cuvette was filled with a total volume of 3.8 ml of 10 mM potassium phosphate (pH 6.6), 50 mM KCl, 0.05% DDM (*n*-dodecyl β-D-maltoside; Melford Laboratories), 2 mM sodium ascorbate, 40 μM TMPD (*N*,*N*,*N*′,*N*′-tetramethyl-*p*-phenylenediamine, a redox mediator), 30 μM total myoglobin and mitos to give 1–3 nM C*c*O. After the baseline had stabilized, the reaction was initiated with 50 μM horse heart cyt *c*. The reaction was complete in 10–15 min. The ratio of oxyferrous/ferrous myoglobin was used to determine both free oxygen concentrations and rates of oxygen consumption, using an oxygen dissociation constant of 1.34 μM [37]. These values were used to determine the *K*_m_ of C*c*O for oxygen by non-linear fitting of data to the Michaelis–Menten equation using the curve-fitting toolbox in Matlab™.

### Determination of turnover numbers and kinetic parameters for cyt *c*

Steady-state oxygen consumption rates were measured in a stirred reaction vessel of a Clark-type O_2_ electrode at 25°C. Assays with whole cells were carried out using 12.5 mg/ml cells giving 10–25 nM C*c*O in 50 mM potassium phosphate (pH 7.2), 440 mM sucrose, 10 mM lactate, 1.8 μM CCCP (carbonyl cyanide *m*-chlorophenylhydrazone) and 1 μM valinomycin [38]. Assays with mitos were carried out using membranes containing 2–10 nM C*c*O in 10 mM potassium phosphate, pH 6.6, 50 mM KCl, 0.05% DDM, 2 mM sodium ascorbate and 40 μM TMPD. The assay conditions for wild-type yeast C*c*O oxidizing horse heart cyt *c* were optimized in WTCOX5A mitos for pH (range 5.8–7.4), ionic strength (using 0–130 mM KCl), DDM concentration (0–0.2%) and horse heart cyt *c* concentration. The optimum assay conditions were pH 6.6, 0.05% DDM and 50 mM KCl. Under these conditions 50 μM horse heart cyt *c* produced rates that were close to *V*_max_. These conditions were assumed to be also optimal for yeast iso-1 and iso-2 cyts *c*. Initially, a baseline was measured in the absence of cyt *c* and then the reaction was initiated by addition of 50 μM cyt *c*. Turnover numbers and rates are expressed in terms of the number of electrons transferred from cyt *c* per s per C*c*O (e·s^−1^). Rates at each cyt *c* concentration were averaged from two to four repeats. *V*_max_ and *K*_m_ values were determined using non-linear fitting of the Michaelis–Menten equation for single phase kinetics and with the sum of two Michaelis–Menten terms for plots that displayed biphasic kinetics using the curve-fitting toolbox in Matlab™.

## RESULTS

### Cell growth and C*c*O expression levels

All of the mutant strains constructed and analysed in the present study are listed in Table 1. The *COX5B* gene at its genomic locus was not deleted in these mutants since its expression relies on hypoxic conditions and hence is not expressed under the aerobic growth conditions used in the present study. Thus the *COX5A*-deleted strain could not grow on respiratory medium (YPG). In order to compare the catalytic activity of 5A and 5B isoen-zymes in the *COX5A*-deleted strain, we replaced *COX5A* by *COX5B* both in the *COX5A* genomic locus and on a centromeric plasmid. In both cases, *COX5B* expression was under the control of the *COX5A* promoter, the 5B isoenzyme was expressed at normal levels under aerobic growth conditions, and the cells were respiratory growth competent. When the deletion of *COX5A* was combined with deletion of *ROX1*, encoding a transcription repressor of *COX5B*, the resulting mutant was also respiratory growth competent, as previously reported, although the growth in respiratory medium was slower than the wild-type control.

Strains were grown to late exponential phase (log*D*=0.9–1) in YPGal medium for 14–16 h at 28°C. All displayed a wild-type doubling time of 2.7–3.0 h. This yielded 11 g of wet mass of cells per litre of culture for all strains except ΔROX1ΔCOX5A, which yielded ~6 g. The difference in biomass was most probably due to the difference in respiration/fermentation ratio of ΔROX1ΔCOX5A when grown to late exponential phase in galactose medium. The doubling times were measured during early exponential phase where the strains might use more fermentation than respiration, thus no difference in doubling times could be observed between strains.

Reduced minus oxidized visible absorbance difference spectra of whole cells confirmed the presence of C*c*O at approximately 2 nmol of C*c*O per g of wet weight cells in COX5B, pCOX5B, ΔROX1ΔCOX5ApCOX5A and ΔROX1ΔCOX5ApCOX5B, consistent with the level in the WTCOX5A strain. The C*c*O level in ΔROX1ΔCOX5A cells was significantly less than 1 nmol·g^−1^, although difficult to quantify accurately in whole cell spectra.

**Figure 1:**
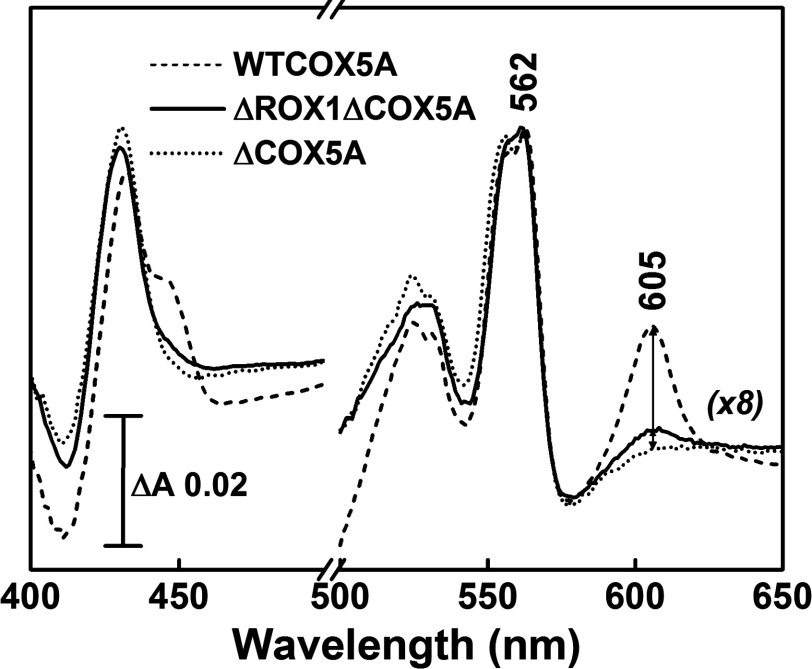
Dithionite reduced minus oxidized visible difference spectra of WTCOX5A (dashed), ΔROX1ΔCOX5A (continuous) and ΔCOX5A (dotted) mitos preparations Mitos were diluted in 50 mM potassium phosphate (pH 7.4) and 2 mM EDTA. 605 nm band is of C*c*O and 562 nm band is of the *bc*_1_ complex. Spectra illustrate a ~6-fold smaller C*c*O/*bc*_1_ ratio in ΔROX1ΔCOX5A. ΔCOX5A lacks C*c*O. The spectra in the 500–700 nm range have been expanded 8-fold for clarity.

Redox spectra of mitos allowed accurate quantification of C*c*O in all strains from either the visible band at 605–621 nm or the Soret band at 445–465 nm [32]. For comparison, Figure 1 includes a spectrum of ΔCOX5A mitos which have no assembled C*c*O. Mitos from WTCOX5A, COX5B, pCOX5B, ΔROX1ΔCOX5ApCOX5A and ΔROX1ΔCOX5ApCOX5B have approximately equivalent levels of C*c*O (0.23–0.31 nmol per mg of mitochondrial protein; nmol of C*c*O·mg^−1^), whereas mitos from ΔROX1ΔCOX5A contained 5–6-fold less C*c*O (0.05±0.01 nmol·mg^−1^). However, the level of *bc*_1_ complex remained fairly constant in mitos from all strains, including those derived from ΔROX1ΔCOX5A, at 0.76–1.2 nmol of *bc*_1_ complex·mg^−1^ (as measured from the visible band at 562–575 nm using an Δ*ε* of 28 mM^−1^·cm^−1^) (Figure 1) [32]. Hence the ratio of C*c*O to *bc*_1_ complex in ΔROX1ΔCOX5A mitos was also ~6-fold less (~0.04:1) than the ratio (0.25:1) in mitos derived from strains WTCOX5A, COX5B, pCOX5B, ΔROX1ΔCOX5ApCOX5A and ΔROX1ΔCOX5ApCOX5B.

### Turnover numbers in whole cells

**Figure 2:**
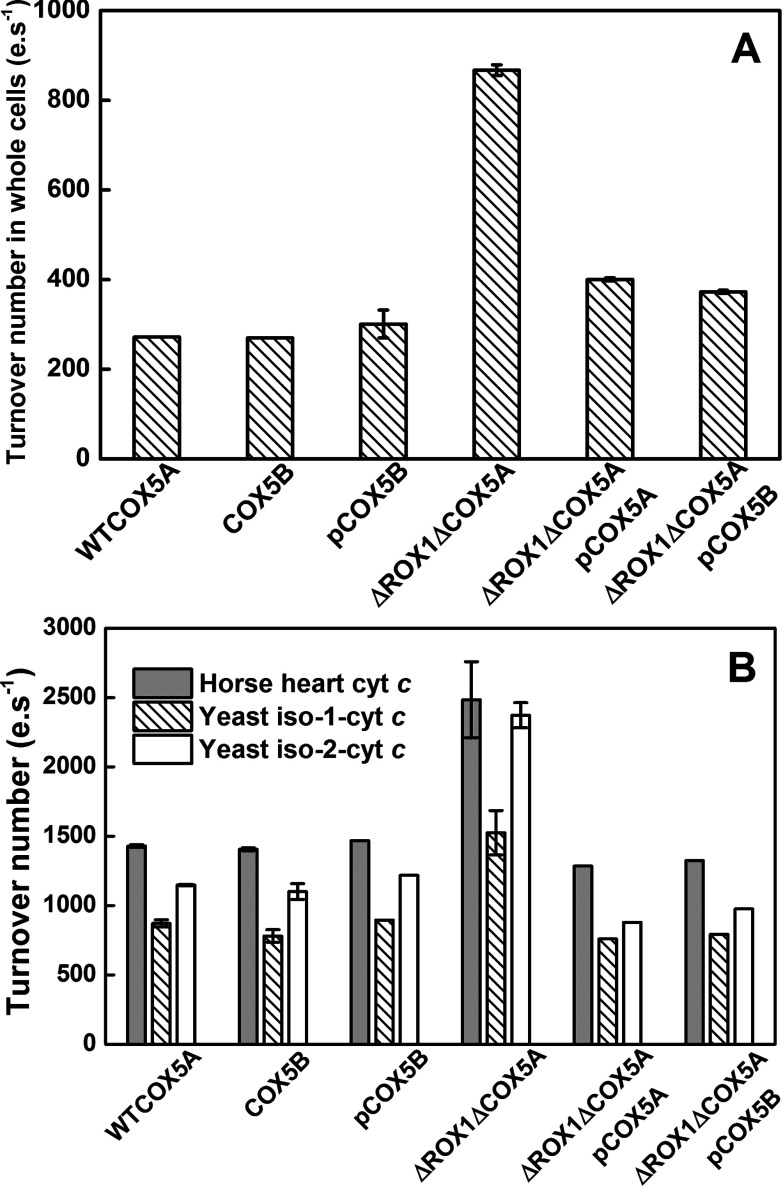
Turnover numbers of yeast C*c*O strains in (A) whole cells and (B) mitos preparations using horse heart cyt *c* (grey), yeast iso-1 cyt *c* (hatched) and yeast iso-2 cyt *c* (white) as substrate (**A**) In whole cells turnover numbers were measured using an oxygen electrode with 12.5 mg/ml cells containing 10–25 nM C*c*O in 440 mM sucrose, 50 mM potassium phosphate (pH 7.2), 10 mM lactate, 1.8 μM CCCP and 1 μM valinomycin at 25°C. (**B**) In mitos preparations turnover numbers were measured using 10–50 μl of mitos containing 2–10 nM C*c*O in 10 mM potassium phosphate (pH 6.6), 50 mM KCl, 0.05% DDM, 40 μM TMPD and 2 mM sodium ascorbate at 25°C and 1 ml final volumes. Once the baseline had stabilized 50 μM cyt *c* was added to initiate the reaction. Results are means±S.D. for two to four repeats.

The steady-state turnover numbers of C*c*O in whole cells under uncoupled conditions are shown in Figure 2(A). Strains that expressed the 5B isoenzyme under the control of the *COX5A* promoter from either the nuclear genome (strain COX5B) or from a plasmid (pCOX5B and ΔROX1ΔCOX5ApCOX5B) had turnover numbers in the same range as the 5A isoenzyme (~330 e·s^−1^) that had been expressed either in wild-type cells or from a plasmid (ΔROX1ΔCOX5ApCOX5A). However, when the 5B isoenzyme was expressed under its own promoter after deletion of *ROX1* in the ΔROX1ΔCOX5A strain, it displayed a turnover number at least 2.6-fold higher, confirming the prior literature reports [29]. The activity of C*c*O of ΔROX1ΔCOX5A expressed as e·s^−1^·g^−1^ of wet weight of whole cells was also greater (~1.4-fold).

### Turnover numbers in mitos preparations

The uncoupled turnover numbers of C*c*O in whole cells will most probably be limited by additional factors, and so turnover numbers in isolated mitos were also compared using conditions that had been optimized for oxidation of horse heart cyt *c* (Figure 2B). In addition, turnover numbers were determined using yeast iso-1 cyt *c* and iso-2 cyt *c* under the same conditions (see the Discussion section). For all strains under these conditions, activities were fastest with horse heart cyt *c*, followed by yeast iso-2 cyt *c*. In agreement with whole cell turnover values, C*c*O in mitos isolated from the ΔROX1ΔCOX5A strain had an approximately 2-fold greater turnover number compared with that in all other strains, irrespective of the cyt *c* substrate used (Figure 2B). However, the C*c*O activity per mg of mitochondrial protein was ~3-fold less than that in the other strains (140 e·s^−1^·mg^−1^ compared with 380 e·s^−1^·mg^−1^) because of the lower level of C*c*O per mg of protein.

### Determination of *K*_m_ for oxygen

Rates of oxygen consumption in the concentration range of the *K*_m_ for oxygen of C*c*O were determined by the oxymyoglobin method [35]. *K*_m_ and *V*_max_ values for WTCOX5A, COX5B and ΔROX1ΔCOX5A mitos are summarized in Table 2 and Michaelis–Menten plots are shown in the Supplementary Figure S1. All strains exhibited approximately the same *K*_m_, although the *V*_max_ of ΔROX1ΔCOX5A was at least 1.5-fold greater compared with that of WTCOX5A and COX5B.

**Table 2:** Comparison of oxygen affinities and *V*_max_ of WTCOX5A, COX5B and ΔROX1ΔCOX5A mitos The *K*_m_ and *V*_max_ values were determined using non-linear fitting of the Michaelis–Menten equation to plots shown in Supplementary Figure S1. Error values are 95% confidence intervals given by the *t*-distribution.

Substrate	Strain (mitos)	*V*_max_ (μM O_2_/s per μM C*c*O)	*K*_m_ (μM)
Oxygen	WTCOX5A	184±12	0.85±0.1
	COX5B	154±9	0.74±0.1
	ΔROX1ΔCOX5A	302±21	0.86±0.2

### *K*_m_ and *V*_max_ for cyt *c*

**Figure 3:**
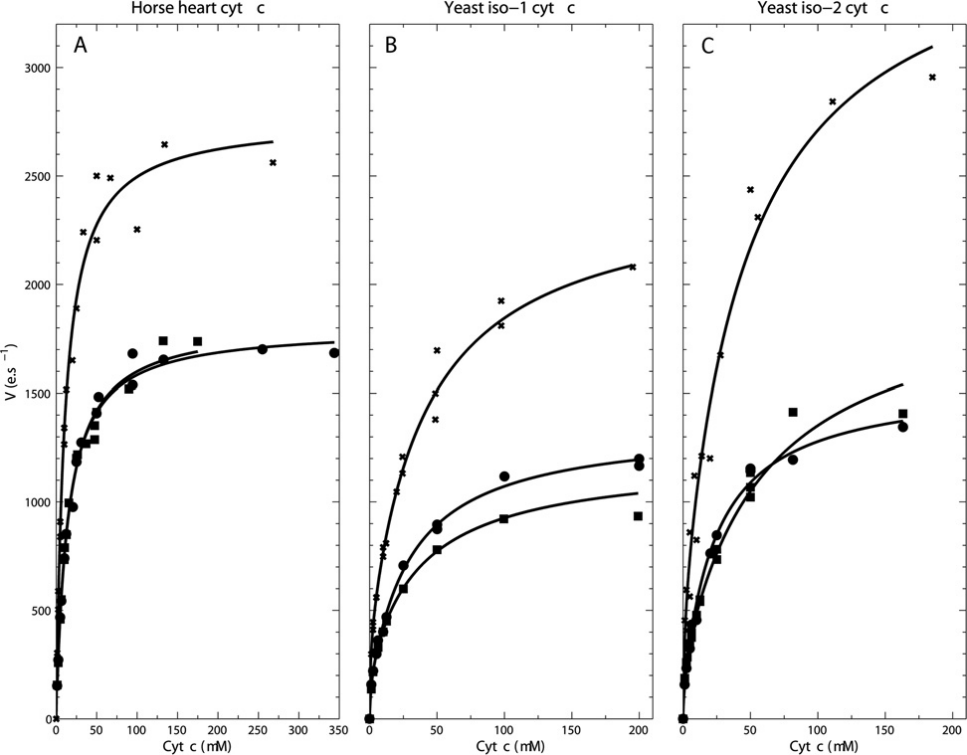
Michaelis–Menten plots of WTCOX5A (●), COX5B (■) and ΔROX1ΔCOX5A (×) mitos using horse heart cyt *c* (A), yeast iso-1 cyt *c* (B) and yeast iso-2 cyt *c* (C) Reaction conditions were the same as in Figure 2(B), but the rate was measured at multiple cyt *c* concentrations (1.25–350 μM). *V*_max_ and *K*_m_ values (see Table 3) were determined using non-linear fitting of the Michaelis–Menten equation for data displaying single phase kinetics (**A**) and two Michaelis–Menten terms for those with biphasic kinetics (**B** and **C**).

Michaelis–Menten plots for WTCOX5A, COX5B and ΔROX1ΔCOX5A mitos are shown in Figure 3 and the derived *V*_max_ and *K*_m_ values for horse heart, yeast iso-1 and iso-2 cyts *c* are summarized in Table 3. Under these assay conditions, the data displayed monophasic kinetics for horse heart cyt *c* but biphasic kinetics for yeast iso-1 and iso-2 cyt *c*. Eadie–Hofstee plots of the data and the fits are shown in Supplementary Figure S2 as these illustrate the mono- or bi-phasic kinetic behaviour more clearly. WTCOX5A and COX5B displayed the same *V*_max_ and *K*_m_ values irrespective of the cyt *c* source, whereas the ΔROX1ΔCOX5A strain again had a *V*_max_ 1.5–2-fold greater, but with a similar *K*_m_.

**Table 3:** Comparison of *V*_max_ and *K*_m_ of WTCOX5A, COX5B and ΔROX1ΔCOX5A mitos for horse heart cyt *c*, yeast iso-1 cyt *c* and iso-2 cyt *c* The *V*_max_ and *K*_m_ values were determined using non-linear fitting of a single Michaelis–Menten equation for assays with horse heart cyt *c* and with two Michaelis–Menten terms for assays with yeast iso-1 and iso-2 cyt *c* that displayed biphasic kinetics (Figure 3). Error values are 95% confidence intervals given by the *t*-distribution.

Substrate	Strain (mitos)	Low-affinity phase	
		*V*_max_ (e·s^−1^)	*K*_m_ (μM)
Horse heart cyt *c*	WTCOX5A	1803±50	13.9±2
	COX5B	1831±80	14.5±2
	ΔROX1ΔCOX5A	2764±140	10.8±2
Yeast iso-1 cyt *c*	WTCOX5A	1231±100	29.8±11
	COX5B	951±120	39.5±19
	ΔROX1ΔCOX5A	2060±200	41.2±18
Yeast iso-2 cyt *c*	WTCOX5A	1448±140	23.9±7
	COX5B	1765±180	59.5±7
	ΔROX1ΔCOX5A	3238±660	47.8±24

## DISCUSSION

Incorporation of subunit 5 (A or B) is essential for the stable expression of optically detectable functional C*c*O [20,39] and hence the level of detectable C*c*O is a direct measure of the level of functionally incorporated subunit 5. It has also been shown that the COX5B isoform is not expressed under aerobic growth conditions [24]. Hence, for those strains used to generate the 5A isoenzyme of C*c*O, the optically detected C*c*O will have only subunit 5A since they were grown aerobically. For those strains used to generate the 5B isoenzyme, 5A isoenzyme was absent since the *COX5A* gene was replaced entirely by *COX5B*.

*COX5A* deletion (Δ*cox5A*) results in a lack of respiratory competence [24] and absence of C*c*O (Figure 1). Respiration-competent mutants of such a Δ*cox5A* strain had mutations in *ROX1*, a repressor of *COX5B*, that resulted in the enhanced expression of *COX5B* [24]. The 5B isoenzyme was up to 3-fold more catalytically active than the 5A isoenzyme. The results of the present study with the ΔROX1ΔCOX5A mutant confirm this observation. A 2-fold faster 5B isoenzyme activity was also observed in a *COX5A*-deleted strain with wild-type *ROX1* and with *COX5B* cloned on a high copy plasmid [30]. However, in all of these strains, the level of C*c*O was significantly diminished in comparison with wild-type levels: 34% of the control in the strain with both Δ*cox5A* and *rox1* mutations [29]; and less than 50% in the Δ*cox5A* strain with higher copy number of *COX5B* [30]. It was also substantially decreased in the ΔROX1ΔCOX5A strain described in the present study.

In order to circumvent such complications, we generated *COX5A*-deleted mutants in which the *COX5B* was placed downstream of the *COX5A* promoter on the genomic locus, similarly to the chimaeric constructs described in [39]. In addition, *COX5A*-deleted strains with or without *ROX1* deletion were transformed with a centromeric plasmid to express 5A or 5B isoforms under the control of the *COX5A* promoter. This enabled expression of 5A or 5B isoforms at wild-type levels and at normal oxygen concentrations and with the same status (present or deleted) of *ROX1*. In these constructs, 5A and 5B isoenzymes failed to show any difference in their *K*_m_ values for oxygen (~0.8±0.1 μM), that are in a similar range to those (1 μM [40] and 0.95 μM [41]) reported for bovine C*c*O. This latter comparison was carried out since their expression is differentially regulated by oxygen concentration. Similarly, the *V*_max_ and *K*_m_ values for cyt *c* from horse heart showed no differences between WTCOX5A and COX5B but were ~1.5-2-fold greater in *V*_max_ in the 5B isoenzymes of C*c*O expressed in the ΔROX1ΔCOX5A strain. The *V*_max_ and *K*_m_ values of WTCOX5A with horse heart cyt *c* reported in the present study are in a range consistent with Geier et al. [42] (*V*_max_ 1773 e·s^−1^, *K*_m_ 15.3 μM).

Equivalent *V*_max_ and *K*_m_ measurements were made using *S. cerevisiae* cyt *c* (Figure 2B and Table 3) since two isoforms are differentially expressed depending on the oxygen levels. Iso-1 cyt *c* is predominantly expressed under normal oxygen levels and iso-2 cyt *c* under hypoxic conditions [25,26]. Once again no significant difference in *V*_max_ was observed between WTCOX5A and COX5B (Figure 2B and Table 3). The *V*_max_ and *K*_m_ values with yeast iso-1 and iso-2 cyt *c* are much greater than those reported (*V*_max_ < 40 s^−1^; *K*_m_ < 10 μM; [28]). Apart from the overall assay conditions and possibly the method used to prepare mitos, these differences in *V*_max_ and *K*_m_ could be due to the higher and more physiologically relevant (0.5 mM cyt *c* [43]) cyt *c* concentration range used in the present study (~300–1.25 μM) compared with the range (20–0.05 μM) used by Allen et al. [28]. WTCOX5A, COX5B and ΔROX1ΔCOX5A strains shared the same *K*_m_ values for cyt *c* (Table 3), as was also observed by Allen et al. [28].

Noticeably, for all strains, the *V*_max_ values were in the order: horse heart > iso-2 > iso-1 cyt *c* (Figure 2B and Table 3). This finding conflicts with the previous report of faster activity with iso-1 cyt *c* compared with iso-2 cyt *c* [28]. However, this is probably accounted for by the different conditions of measurement, which in the present study were optimized in pH and ionic strength for horse heart cyt *c*. More significantly, it was reported that the isoenzymes showed different relative rates with the cyt *c* isoforms, with selectivity ‘dampened’ from 4-fold to 1.6-fold when the physiologically relevant aerobic (C*c*O isoenzyme 5A/iso-1 cyt *c*) and hypoxic (C*c*O isoenzyme 5B/iso-2 cyt *c*) isoforms were paired together [28]. However, no preferences for specific cyt *c* isoforms were evident in the 5A and 5B isoenzymes in the present study and, if anything, the opposite effect was observed when comparing WTCOX5A/iso-1 cyt *c* and ΔROX1ΔCOX5A/iso-2 cyt *c* pairs (Figure 2B and Table 3).

Biphasic kinetics (Figure 3 and Supplementary Figure S2) were observed for yeast cyt *c*, consistent with previous data [28], whereas horse heart cyt *c* exhibits single phase kinetics consistent with the findings in [42]. The phenomenon has been well-documented for many types of C*c*O and with different types of cyts *c* (including yeast C*c*O) [44,45]. Several models have been proposed to explain the biphasic behaviour. One model is that there are two catalytic sites on C*c*O that can bind cyt *c* with different affinities [44]. A second proposal is that there is a non-catalytic binding site for cyt *c*, either very close to the catalytic site and so allowing direct interaction with substrate [46], or at a distance and acting allosterically [47]. The transition of high-affinity to low-affinity phase, was suggested to arise from an increase in the dissociation rate constant of ferricyt *c* (the rate-limiting step) when the regulatory site was bound by a second cyt *c*. Most recently, it was proposed that oxidized cyt *c* also acts as a competitive inhibitor of C*c*O, which is in addition to the effects of binding of cyt *c* to a second regulatory site [48]. For the purpose of the present study, a comparison of WTCOX5A, COX5B and ΔROX1ΔCOX5A isoenzymes has been made with the high-velocity/low-affinity phase data since it is the phase that may predominate under physiological conditions since cyt *c* concentration in the intermembrane space is at 0.5 mM [43] and it is observed for horse heart, yeast iso-1 and iso-2 cyt *c* (Table 3).

To summarize, yeast strains that express the 5A or 5B isoenzymes to the same levels under aerobic growth conditions, with or without the deletion of the *ROX1* gene, show definitively that these isoenzymes display similar turnover numbers and affinities for oxygen and cyt *c*. Hence the origin of the elevated activity of the 5B isoenzyme reported previously [28,29] and confirmed in the present study with the equivalent ΔROX1ΔCOX5A strain must be caused by a secondary effect. Two possible explanations are discussed in the present paper.

Rox1 is a transcription repressor of many hypoxic genes [49,50]. It is possible that its loss could cause increased 5B isoenzyme activity through separate effects that might lead to post-translational modification of subunit 5B, for example phosphorylation or binding/unbinding of ATP/ADP [4,51]. However, elevated 5B isoenzyme activity has been previously observed when expressed from a high copy number plasmid both in the presence of *ROX1* [30] and in a *rox1*-mutated strain [28]. Furthermore, in the present study the 5B isoenzyme had the same activity (and expression level) as the 5A isoenzyme when expressed both with (in the COX5B strain) and without (in the ΔROX1ΔCOX5ApCOX5B strain) a functioning Rox1 (Figure 1). All of the above argues against a secondary effect of *ROX1* deletion as the prime cause of the increased activity of the 5B isoenzyme when produced at low oxygen tensions or in strains such as ΔROX1ΔCOX5A.

Elevated activity of the 5B isoenzyme has been observed only when its expression level was significantly lower than in wild-type strains, as is also the case for the ΔROX1ΔCOX5A strain reported. Since the level of *bc*_1_ complex remains relatively constant, this results in a proportionately lower C*c*O/*bc*_1_ ratio. It has been shown that a proportion of C*c*O in yeast mitochondria can be isolated as C*c*O–*bc*_1_ supercomplexes [52–54]; the proportion of C*c*O in such supercomplexes will presumably be governed by the C*c*O/*bc*_1_ ratio. It seems feasible that the interaction between complexes may induce allosteric effects that elevate the catalytic activity of C*c*O, an effect that becomes more evident when the C*c*O/*bc*_1_ ratio is low since this will favour a greater proportion of the C*c*O forming C*c*O–*bc*_1_ supercomplexes (simply from an equilibrium constant point of view). Interestingly, the III_2_IV_2_ supercomplex structure of yeast C*c*O shows that subunit 5 is close to the interface with the *bc*_1_ complex [54]. The extent of supercomplex formation, and the allosteric effect that it induces, could be different with the 5A and 5B isoforms, providing a novel possible functional basis for the existence of the isoenzymes.

## Online data

Supplementary data
