# Supplementary material for: Comparisons of subunit 5A and 5B isoenzymes of yeast cytochrome c oxidase
Source: Biochem J. 2014 Dec 5;464(Pt 3):335–42. doi: 10.1042/BJ20140732 (PMC4255728; doi:10.1042/BJ20140732)
Supplement: Supplementary data [file bj4640335ntsadd.pdf]

SUPPLEMENTARY DATA

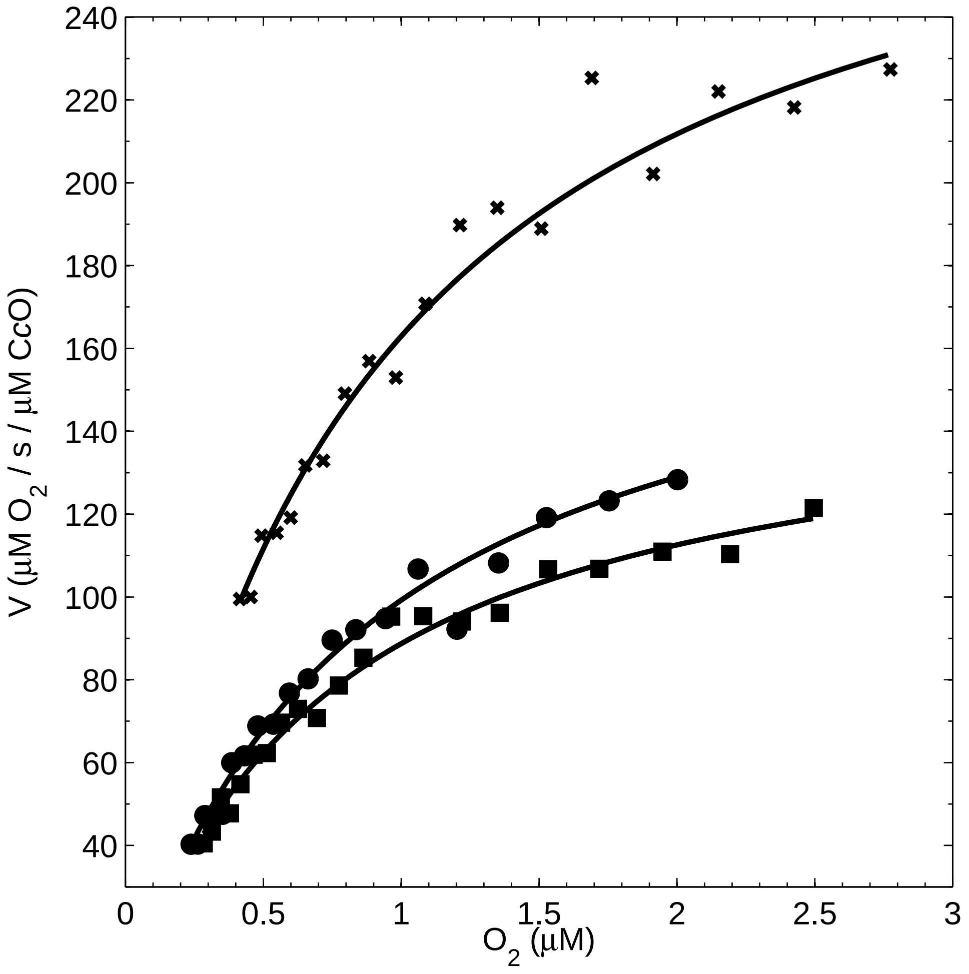

Figure S1 Michaelis-Menten plots derived by following the deoxygenation of horse heart oxymyoglobin by mitos preparations of yeast WTCOX5A (●), COX5B (■) and ΔROX1ΔCOX5A (x)

Assay was carried out in 10 mM potassium phosphate at pH 6.6, 50 mM KCl, 0.05 % (w/v) DDM, 2 mM sodium ascorbate, 40 μM TMPD, 30 μM total horse heart myoglobin and mitos to give 1-3 nM C*c*O. After the baseline had stabilised the reaction was initiated with 50 μM horse heart cyt *c*. V_{max} and K_m values were determined using non-linear fitting of the Michaelis-Menten equation and are summarised in Table 2.

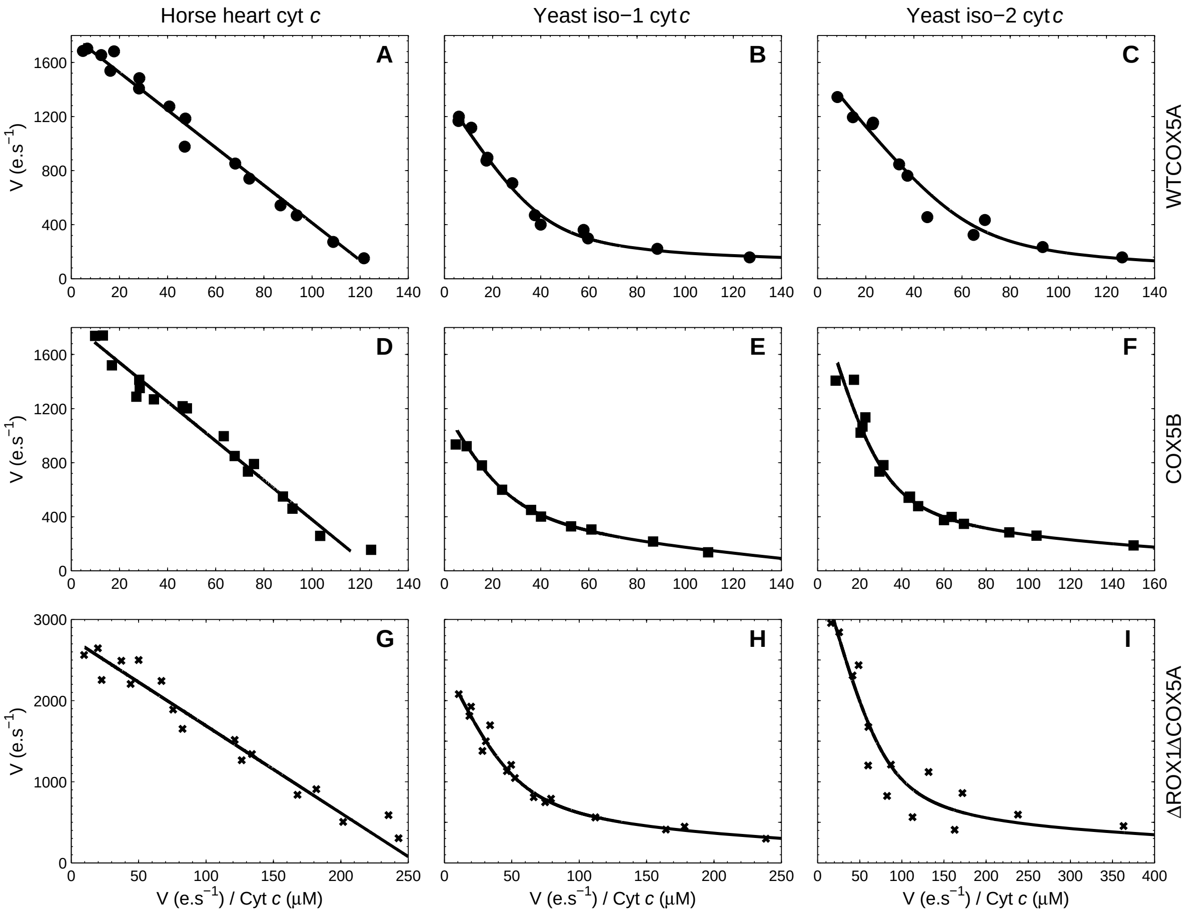

Figure S2 Eadie-Hofstee plots of WTCOX5A (●), COX5B (■) and ΔROX1ΔCOX5A (x) mitos using horse heart cyt *c* (A, D, G), yeast iso-1 cyt *c* (B, E, H) and yeast iso-2 cyt *c* (C, F, I)

Eadie-Hofstee transformation of the non-linear regression is also overlaid. Turnover numbers were measured at multiple cyt *c* concentrations (1.25-400 μM) using mitos containing 2-10 nM C*c*O in 10 mM potassium phosphate, 50 mM KCl, pH 6.60, 0.05 % (w/v) DDM, 40 μM TMPD, 2 mM sodium ascorbate at 25 ^{o}C.

Table S1 Comparison of V_{max} and K_m of WTCOX5A, COX5B and ΔROX1ΔCOX5A mitos for horse heart cyt *c*, yeast iso-1 cyt *c* and yeast iso-2 cyt *c*

The V_{max} and K_m values were determined using non-linear fitting of the Michaelis-Menten equation for all single phase kinetics and with two Michaelis-Menten terms for plots that displayed biphasic kinetics (see Figure 3 and Supplementary Figure S2). Error values are 95 % confidence intervals given by the *t*-distribution.

Substrate	Strain (mitos)	Low affinity phase		High affinity phase	
		V_{max} ($e.s^{-1}$)	K_m (μM)	V_{max} ($e.s^{-1}$)	K_m (μM)
Horse heart cyt *c*	WTCOX5A	1803 ± 50	13.9 ± 2	-	-
	COX5B	1831 ± 80	14.5 ± 2	-	-
	ΔROX1ΔCOX5A	2764 ± 140	10.8 ± 2	-	-
Yeast Iso-1 cyt *c*	WTCOX5A	1231± 100	29.8 ± 11	123.8 ± 120	0.11 ± 2
	COX5B	951 ± 120	39.5 ± 19	247.8 ± 150	1.48 ± 2
	ΔROX1ΔCOX5A	2060 ± 200	41.2 ± 18	388.5 ± 250	2.04 ± 0.3
Yeast Iso-2 cyt *c*	WTCOX5A	1448 ± 140	23.9 ± 7	110 *	0.5 ± 2
	COX5B	1765 ± 180	59.5 ± 7	247.3 ± 160	0.8 ± 2
	ΔROX1ΔCOX5A	3238 ± 660	47.8 ± 24	526 *	1.1 ± 2

*fits were based on fixing one variable (V_{max} of high affinity phase) to a value optimised based on the R-squared.
